# Complementary feeding practices among children in Benishangul Gumuz Region, Ethiopia

**DOI:** 10.1186/s13104-017-2663-0

**Published:** 2017-07-27

**Authors:** Dula Ayana, Amare Tariku, Amsalu Feleke, Haile Woldie

**Affiliations:** 1 Department of Nursing, Pawe Health Science College, Pawe District, Ethiopia; 2 Department of Human Nutrition, Institute of Public Health, College of Medicine and Health Sciences, University of Gondar, Gondar, Ethiopia; 3 Department of Health Service Management and Heath Economics, Institute of Public Health, College of Medicine and Health Sciences, University of Gondar, Gondar, Ethiopia

**Keywords:** Complementary feeding practice, Children aged 6–23 months, Ethiopia

## Abstract

**Background:**

Appropriate complementary feeding helps to reduces child’s risk of undernutrition, infectious disease and related mortality. However, complementary feeding practices are sub-optimal in Ethiopia. There is, however, also limited evidence in the country, particularly of Pawie District. Therefore, this study aimed to assess timely initiation of complementary feeding and associated factors among mothers who had children aged 6–23 months in Pawie District, Benishangul Gumuz Regional State.

**Methods:**

A community based cross-sectional study was conducted in Pawie District from February 01 to March 29, 2015. A multi-stage sampling technique was employed to select 806 mother–child pairs. Multivariable logistic regression analysis was used to investigate factors associated with timely initiation of complementary feeding. Adjusted odds ratio (AOR) with corresponding 95% Confidence Interval was calculated to show the strength of association. A p value of <0.05 was used to declare significance of association.

**Results:**

The overall prevalence of timely initiation of complementary feeding was 61.8%. One quarter (23.7%) of children had good dietary diversity and 32.7% of children aged 12–23 months were fed with appropriate meal frequency. Mother’s place of residence: urban settlement [AOR = 2.11, 95% CI 1.47, 3.02] and postnatal checkup [AOR = 1.68, 95% CI 1.15, 2.45] were significantly associated with timely initiation of complementary feeding.

**Conclusions:**

The prevalence of timely initiation of complementary feeding was low in Pawie District. Therefore, further strengthening maternal postnatal care utilization is a key to improve timely initiation of complementary feeding. Moreover, attention needs to be given to the rural mothers.

## Background

The first 2 years of life are critical window to promote optimal child growth and development of the child [1]. After the age of sixth month, the energy and nutrient content of breast milk alone is not enough to meet nutritional demand of the growing infant. Therefore, initiation of complementary feeding, defined as process of starting additional foods and liquids along with breast milk, is essential to ensure optimal catch-up growth [2]. To this effect, the World Health Organization (WHO) recommends to initiate nutritionally adequate, safe, and appropriate complementary food at the age of sixth month [3]. Optimal complementary feeding (CF) helps to reduces child’s risk of acquisition of different infectious diseases and related mortality [4–6]. Also, it improves the child’s mental and motor development, and protects against obesity and other metabolic diseases later in life [7–11].

Despite the enormous benefit of appropriate complementary feeding, only 35% of infants worldwide have a timely initiation of CF [12]. In Asia, for instance, the median age of introducing additional food ranges from 3.8 months in China to 5.5 months in Japan and Maldives [13]. Similarly, according to the former studies in Africa less than half of infants start CF at the recommended age of 6 months [14–18]. In Ethiopia, the 2011 Demographic and Health Survey (EDHS) report indicated that only 49% of infants aged 6–8 months were given any complementary food. Surprisingly, only 4% of children aged 6–23 months were found having appropriate infant and young child feeding (IYCF) practice [19].

Inappropriate feeding practice is associated with the adverse and multi-dimensional health and developmental consequences. It causes more than two-third of under-five child mortality [1, 12], in which 41% of these deaths occur in Sub-Saharan Africa [20]. In addition, any damage caused by nutritional deficiencies in the early childhood is related to impaired cognitive development, poor educational achievement and low economic productivity [4, 7, 21]. Inappropriate CF is also associated with child undernutrition [4, 22].

According to studies conducted elsewhere, maternal socio-demographic and health care related characteristics are the significant factors associated with timely initiation of CF. Married, housewives, unemployed, and multi-parous mothers have a higher likelihood of timely initiation of CF [13–17, 23]. Similarly, antenatal care, postnatal checkup, institutional delivery, and better health care access [24, 25] are positively correlated with timely initiation of CF. However, mothers with a rural residence, low child feeding knowledge, perceived inadequate breast milk production, maternal and paternal illiteracy, and child sex (male) were inversely associated with timely initiation of CF [26–28].

Ethiopia designed different programs and strategies to improve child feeding practices and nutritional status [29–31]. However, inappropriate child feeding practices and undernutrition remains a public health problem [19]. In Ethiopia, most former research on CF practices were confined to urban areas [23, 24, 27, 28, 32–34], however majority of the population reside in rural settlements where poor health care access and illiteracy rate is higher [19]. On the other hand, literature is limited in the Benishangul Gumuz Regional State. Therefore, this study aimed to investigate timely initiation of CF and associated factors among mothers who had children aged 6–23 months in Pawe District, Benishangul Gumuz Regional State, northwest Ethiopia. The finding of this study provides information for program designers and implementers to make evidence based decision to enhance complementary feeding practices.

## Methods

### Study design and setting

A community based cross–sectional study was conducted in Pawe District from February 01 to March 29, 2015. The district is one of the seven districts in Metekel Zone, Benishangul Gumuz Regional State, northwest Ethiopia. It lies at an area of 5244 square kilometers, and located 623 km from the capital city of Ethiopia, Addis Ababa. Administratively the district is structured into 21 kebeles (2 urban and 19 rural kebeles, *the smallest administration unit)*. Based on the 2011 district based census, a total of 76,006 people (37,552 females and 38,454 males) live in the Pawe District, of which 6585 were children aged 6–23 months. The district has one general hospital, 4 health centers, and 15 health posts. A mixed farming, crop and livestock production, is the major the livelihood of the population, and chronic food insecurity is one of the critical public health problem in the study area and the region at large.

### Sample size and sampling procedure

All mothers with children aged 6–23 months who lived in Pawe District for at least 6 months were eligible for the study. A single population proportion formula was used to determine sample size by considering the assumptions: expected prevalence of timely initiation of CF in Ethiopia as 49% [19], a 95% level of confidence, 5% margin of error (d) and a design effect of 2. Finally, a sample size of 806 was obtained after adding a 5% non-response rate.

A stratified multi-stage sampling technique was employed to select the study subjects. Following stratification of kebeles into urban and rural, six kebeles (one urban and five rural kebeles) were selected using the lottery method. According to the Health Extension Workers report, a total of 2319 children aged 6–23 months lived in the selected kebeles. Proportional allocation was used to determine the number of children included in the study in each targeted kebeles. A systematic sampling technique was used to select households with an eligible child. For households with more than one study subject, only one was selected using lottery method. When mother–child pairs were not available at the time of data collection three repeated visits were made.

### Data collection tools and procedures

A pretested and structured questionnaires consisting of dietary diversity score (DDS) tool was used to collect data. Twelve Clinical Nurses and three Health Officers were involved as data collector and supervisor, respectively. The English version questionnaire was translated into Amharic, the native language of the study area, then back to English by English language and public health experts to ensure its consistency. The research assistants (data collectors and supervisors) were trained for 2 days about interview techniques prior to data collection. The questionnaire was pre-tested among 40 mother–child pairs in a community with similar socio-demographic profile as the study area. The clarity, acceptability, and applicability of the procedures were evaluated during this pretest.

### Operational definition and study variables

Complementary feeding practices were assessed according to the WHO recommendation [3]. Accordingly, to ascertain timely initiation of CF, a mother was asked to report the initial time she gave extra food to her child. She was asked as “When did you first introduce any solid, semi-solid or fluid to [child name] in addition to breast milk”.

The standardized DDS tool with 24-h recall was used to qualitatively assess the dietary intake of children. Mothers were interviewed to list the food items consumed by the child in the previous 24 h preceding the date of survey. The food items were categorized into seven food groups as grains, roots and tubers; legumes and nuts; dairy products; flesh foods (meat, fish, poultry and organ meats); egg; vitamin-A rich fruits and vegetables; and other fruits and vegetables [34]. Considering the standardized minimum acceptable DDS [5], a child with DDS of ≥4 was categorized as having good dietary diversity, while participant with a DDS of <4 was deemed to have poor dietary diversity.

Mothers IYCF knowledge was determined using six knowledge item questions which were adopted from the WHO key IYCF indicators [3]. Accordingly, respondents were asked about the health benefit of CF, appropriate time for initiation of additional/complementary food, the minimum acceptable dietary diversity and meal frequency, the dangers of prelacteal feeding, and bottle feeding. Then, if the mothers correctly answer three or more of the above knowledge questions, she was considered as having a good knowledge, otherwise, she had a poor knowledge.

### Data analysis

Data were entered into EPI-info version 3.5.3 and analyzed using the Statistical Package for Social Sciences (SPSS) version 20. Descriptive statistics, including frequencies and proportions, were used to summarize the study variables. A binary logistic regression was used to identify the factors associated with timely initiation of complementary feeding. The bivariable analysis was carried out for all independent variables with the outcome variable, and a p value of <0.2 was used as variable selection criteria. Thus, variables with a p value of <0.2 in the bivariable analysis were fitted into multivariable logistic regression model to control the possible effect of confounders. The strength of association was measured by odds ratios with a 95% confidence interval. Both the crude odds ratio (COR) and adjusted odds ratio (AOR) were reported. Variables with a p value of <0.05 in the multivariable logistic regression model were considered as significant factors.

## Results

### Socio-demographic characteristics

A total of 785 mother–child pairs were included in the study giving a response rate of 97.6%. The mean (±standard deviation, SD) age of the mothers was 30 years (±6.52). Nearly three-fourths (72.5%) of respondents were rural residents. The mean (±SD) family size of the households was 4.8 (±1.6), and about 73.0% of households had ≥5 family members. Most (63.7 and 56.8%, respectively) of the mothers and fathers had no formal education (Table 1).

**Table 1:** Socio-demographic characteristics of children and their parents in Pawe District, northwest Ethiopia, 2015

Characteristics	Frequency	Percent
Place of residence		
Urban	215	27.4
Rural	570	72.6
Sex of the child		
Male	385	49
Female	400	51
Age of the child (in months)		
6–11	251	32.0
12–17	502	63.9
18–23	32	4.1
Birth order of the child		
1st	175	22.3
2nd–3rd	513	65.4
4th and above	97	12.3
Birth interval in years (n = 610)		
No birth interval^a^	175	
1	81	13.3
2–3	394	64.6
≥4	135	22.1
Ethnicity		
Amhara	446	56.7
Agew	96	12.5
Gumuz	71	9.0
Other^b^	172	21.8
Religion		
Orthodox	450	57.3
Muslim	169	21.3
Protestant	146	18.6
Catholic	18	2.3
Family size		
≤5	573	72.9
>5	212	27.1
Number children under 5 years		
One	174	22.2
Two	211	26.8
Three to five	400	51
Mother’s marital status		
Currently married	669	85.2
Currently unmarried	116	14.8
Mother’s educational status		
No formal education	500	63.7
Primary school (1–8)	174	22.2
Secondary school and above	111	14.1
Mother’s employment status		
Housewife	671	85.5
Employed	114	14.5
Father’s educational status		
No formal education	446	56.8
Primary school (1–8)	198	25.2
Secondary school and above	141	18.0
Mother’s age		
15–24	114	(14.5)
25–34	410	(52.2)
35–46	261	(33.3)
Father’s employment status (n = 667)		
Farmer	570	85.4
Other^c^	97	14.7
Possession of TV or radio		
Yes	83	10.7
No	702	89.3
Average monthly income		
<999	584	74.4
1000–2499	113	14.4
≥3000	88	11.2

^a^Children at first birth order or without an elder child

^b^Oromo, Shinasha, Hadiya, Kumbata, and Tigre

^c^Governmental employee and daily laborer

### Mother’s infant and young child feeding knowledge and health care utilization

The majority (93%) of the mothers knew about the negative effects of prelacteal feeding. Three quarter of the mothers knew about the benefit (78%) and the appropriate time to initiate CF (72.0%). However, only 10.7% correctly responded about the minimum dietary diversity. Most of (79.2%) the mothers had postnatal checkup, while 65.1% received counseling about CF during their postnatal visit Table 2).

**Table 2:** Maternal health care and child feeding practices in Pawe District, northwest Ethiopia, 2015

Characteristics	Frequency	Percent
Antenatal care visit		
Yes	445	56.7
No	340	43.3
Number of antenatal care visit (n = 445)		
1–3	238	53.5
≥4	207	46.5
Place of delivery		
Home	537	68.4
Health institution	248	31.6
Postnatal checkup		
Yes	622	79.2
No	163	20.8
Postnatal counseling about CF^*^ (n = 622)		
Yes	405	65.1
No	217	34.9
Mother’s IYCF knowledge		
Good knowledge	422	53.8
Poor knowledge	363	46.2
Ever breast feed		
Yes	761	96.9
No	24	3.1
Initiation of breast feeding (n = 761), h		
≤1	522	68.6
>1	239	31.4
Breast feeding status during the survey (n = 761)		
Yes	665	84.7
No	96	15.3
Prelacteal feeding (n = 704)		
Yes	121	15.9
No	640	84.1
Type of prelacteal food given (n = 122)		
Butter	75	62
Cow milk	73	60.3
Water and sugar	72	59.5
Others^a^	81	67
Bottle feeding		
Yes	318	40.5
No	467	59.5
Initiation of complementary feeding		
Timely initiation (at sixth month)	485	61.8
Early initiation (before sixth month)	249	31.7
Late initiation (after sixth month)	51	6.5
Dietary diversity		
Poor	599	76.3
Good	186	23.7

* Complementary feeding

^a^Formula milk, plain boiled water

### Breastfeeding and complementary feeding practices

Considerably high proportion of (96.9%) children were breastfed at least once in life, and 84.7% were breastfeeding at the time of data collection. More than two-third (68.6%) of the mothers initiated breastfeeding within 1 h after birth. Prelacteal feeding was detected in 15.9% of children (Table 2).

**Fig. 1:**
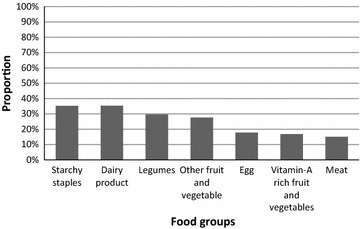
Proportion of children (6–23 months) who consumed individual food groups in the previous 24 h preceding the date of survey, Pawe District, northwest Ethiopia, 2015

From the total interviewed mothers, about 61.8% [95% CI 58.2, 65.4] of them started CF at the infant’s sixth month, whereas about 22.5 and 6.5% of mothers introduced CF before and after the sixth month, respectively. About 67.7, 52.6, and 32.7% of children aged 6–8, 9–11, and 12–23 months fed with the minimum acceptable and age appropriate meal frequency, respectively. One quarter (23.7%) of children had good dietary diversity (Table 2). One-third (35.2 and 35.3%, respectively) of children consumed complementary food made from starchy staples and dairy products. However, only 15% of children ate meat (Fig. 1).

### Factors associated with timely initiation of complementary feeding

The result of bivariable analysis showed that, place of residence, marital status, number of children under 5 years, possession of a TV or radio, and postnatal checkup were significantly associated with timely initiation of CF. However, only place of residence and postnatal checkup were significantly and independently associated with timely initiation of CF. With this regard, the higher odds of timely initiation of CF was noted among mothers who lived in the urban settlements [AOR = 2.11, 95% CI 1.47, 3.02] and had postnatal checkup [AOR = 1.68, 95% CI 1.15, 2.45] (Table 3).

**Table 3:** Factors associated with timely initiation of CF among mothers who had children aged 6–23 months in Pawe District, northwest Ethiopia, 2015

Variables	Timely initiation of CF			
	Yes	No	Crude odds ratio (95% CI)	Adjusted odds ratio (95% CI)
Sex of the child				
Female	247	138	1.00	
Male	238	162	0.82 (0.62, 1.10)	
Place of residence				
Rural	325	245	1.00	1.00
Urban	160	55	2.20 (1.55, 3.11)*	2.11 (1.47, 3.02)*
Mother’s marital status				
Currently married	424	245	1.56 (1.10, 2.32)*	
Currently unmarried	61	55	1.00	
Family size				
≤5 members	364	209	1.31 (0.95, 1.81)	
>5 member	121	91	1.00	
Number of children under 5 years				
1	103	71	0.66 (0.47, 0.94)*	
2	145	66	1.00 (0.70, 1.44)	
3–5	237	163	1.00	
Birth order of index child				
1st	104	71	1.22 (0.74, 2.01)	
2nd–3rd	328	185	0.83 (0.58, 1.18)	
≥4th	53	44	1.00	
Possession of TV or radio				
No	66	17	1.00	
Yes	419	283	0.38 (0.22, 0.66)*	
Postnatal checkup				
Yes	408	214	2.13 (1.5, 3.02)*	1.68 (1.15, 2.45)*
No	77	86	1.00	1.00

* p < 0.05

## Discussion

WHO designed different strategies to achieve optimal implementation of CF practice (≥80%) in the last couple of decades [2, 10, 12, 35]. Ensuring optimal coverage of appropriate CF has special importance for low and middle income countries, including Ethiopia, where majority of children are suffering from undernutrition and related consequences [19, 22].

This study illustrated that, the coverage of timely initiation of CF was 61.8%. The prevalence was slightly higher than the 2011 EDHS report (51%) [19] and other district level reports in Ethiopia: Axum (52.8%) [32] and Kamba (54.4%) [23]. However, the finding was lower than the WHO recommendation for good practice of CF (≥80%) [35] and the study reports of other developing countries, such as India (77.5%) [36] and Nepal (87.3%) [37]. This difference could be explained by higher maternal literacy rate and utilization of institutional delivery in the latter study areas, which are the main fertile grounds to step-up mothers’ confidence in challenging the community attitude towards inappropriate feeding practices. The previous researches also illustrated that mother’s education was positively associated with timely initiation of CF [38–41].

The result of multivariate analysis showed that mothers postnatal checkup enhances the odds of timely initiation of CF. The result was consistent with other reports of both developed and developing countries [23, 42–46]. In fact, postnatal checkup is an important platform to improve mothers’ knowledge and change unfavorable attitude towards implementation of appropriate child feeding practices. These positive effects mainly operate through child feeding counseling and behavioral change and communication interventions [36]. Number of researches affirmed favorable effect of mother’s IYCF knowledge on implementation of appropriate CF practices [47–52].

Furthermore, the increased odds of timely initiation of CF were found among urban mothers compared to the rural dwellers. Similar findings were also reported by the earlier local studies [24, 34, 53]. In Ethiopia, the level of maternal health care utilization varies with residence, in which mothers living in the urban settlements were found with high level of service utilization [19]. These disparities in utilization of basic health cares could ease access to information on appropriate child feeding practices which ultimately improves the likelihood women adherence to appropriate IYCF recommendations [20, 54].

This study showed complementary feeding practice of children in the predominantly rural population of northwest Ethiopia where limited scientific evidences are available. Moreover, efforts, including adequate training and frequent supervision, were made improve the quality of the data. However, the study is not free from some limitations. As an illustration, there is a chance to commit recall bias hence the measurement of some variables (child feeding practice) depends on mother’s recall.

## Conclusions

Complementary feeding practice was sub-optimal in Pawe District. Mothers postnatal checkup and urban residence were significantly associated with timely initiation of complementary feeding. As a result, increasing the coverage of postnatal care utilization is crucial to implement appropriate complementary feeding practices. Standardizing the basic health care elements, IYCF counseling and behavioral change communication interventions, in postnatal care package are also critical in addition to increasing the service utilization. Furthermore, special attention needs to be given for the rural mothers.

## Abbreviations

COR: crude odds ratio
AOR: adjusted odds ratio
WHO: World Health Organization
CF: complementary feeding
EDHS: Ethiopia Demographic and Health Survey
IYCF: infant and young child feeding
DDS: Dietary Diversity Score
SD: standard deviation
TV: television
